# The effect of occipital nerve field stimulation on the descending pain pathway in patients with fibromyalgia: a water PET and EEG imaging study

**DOI:** 10.1186/s12883-018-1190-5

**Published:** 2018-11-12

**Authors:** Shaheen Ahmed, Mark Plazier, Jan Ost, Gaetane Stassijns, Steven Deleye, Sarah Ceyssens, Patrick Dupont, Sigrid Stroobants, Steven Staelens, Dirk De Ridder, Sven Vanneste

**Affiliations:** 1 School of Behavioral and Brain Sciences, The University of Texas at Dallas, Richardson, Texas, USA; 2 Department of Neurosurgery, University Hospital Antwerp, Antwerp, Belgium; 3 BRAI3N, Ghent, Belgium; 4 Department of physical health hand rehabilitation, University Hospital Antwerp, Edegem, Belgium; 5 Department of Cognitive Neurology, UZ Leuven, Leuven, Belgium; 6 Department of nuclear medicine, University Hospital Antwerp, Edegem, Belgium; 7 Molecular Imaging Centre, University of Antwerp, Edegem, Belgium; 8 Department of Surgical Sciences, Dunedin School of Medicine, University of Otago, Dunedin, New Zealand

**Keywords:** Positron emission tomography (PET), Occipital nerve stimulation, Fibromyalgia

## Abstract

**Background:**

Fibromyalgia is a chronic disorder characterized by widespread musculoskeletal pain accompanied by fatigue, sleep, memory, and mood problems. Recently, occipital nerve field stimulation (ONS) has been proposed as an effective potential treatment for fibromyalgia-related pain. The aim of this study is to unravel the neural mechanism behind occipital nerve stimulation’s ability to suppress pain in fibromyalgia patients.

**Materials and methods:**

Seven patients implanted with subcutaneous electrodes in the C2 dermatoma were enrolled for a Positron Emission Tomography (PET) H_2_^15^O activation study. These seven patients were selected from a cohort of 40 patients who were part of a double blind, placebo-controlled study followed by an open label follow up at six months. The H_2_^15^O PET scans were taken during both the “ON” (active stimulation) and “OFF” (stimulating device turned off) conditions. Electroencephalogram (EEG) data were also recorded for the implanted fibromyalgia patients during both the “ON” and “OFF” conditions.

**Results:**

Relative to the “OFF” condition, ONS stimulation resulted in activation in the dorsal lateral prefrontal cortex, comprising the medial pain pathway, the ventral medial prefrontal cortex, and the bilateral anterior cingulate cortex as well as parahippocampal area, the latter two of which comprise the descending pain pathway. Relative deactivation was observed in the left somatosensory cortex, constituting the lateral pain pathway as well as other sensory areas such as the visual and auditory cortex. The EEG results also showed increased activity in the descending pain pathway. The pregenual anterior cingulate cortex extending into the ventral medial prefrontal cortex displayed this increase in the theta, alpha1, alpha2, beta1, and beta2 frequency bands.

**Conclusion:**

PET shows that ONS exerts its effect via activation of the descending pain inhibitory pathway and the lateral pain pathway in fibromyalgia, while EEG shows activation of those cortical areas that could be responsible for descending inhibition system recruitment.

**Trial Registration:**

This study is registered with ClinicalTrials.gov, number NCT00917176 (June 10, 2009).

## Background

Fibromyalgia is a pain syndrome characterized by widespread chronic pain in the four quadrants of the body that can be attributed to abnormalities in central pain processing circuits, rather than to damage to or inflammation of peripheral areas [1]. Fibromyalgia is not restricted to pain symptoms alone but also includes non-restorative sleep, fatigue, headaches, and mood disorders [2]. The American College of Rheumatologists (ACR) proposed diagnostic criteria in 1990. In 2010, these criteria were revised taking into account 18 pain areas and as well as other symptoms such as fatigue, memory disturbances, lower abdominal cramps, depressive mood, and headache as diagnostic criteria. The prevalence of fibromyalgia fluctuates between 0.4% up to 9.4% [3]. The economic burden of this disease is extremely high with a health related costs in a United States population up to o $11,049 per patient per year [4, 5]. A plethora of treatments have been trialed in clinical studies. Current treatment methods consist of both pharmacological (e.g. antidepressants, anti-seizure medication, etc.) and non-pharmacological approaches (e.g. exercise therapy, massage therapy, etc.); however, a group of fibromyalgia patients remains refractory with these treatments [6, 7]. Therefore, improvements to current treatments and new treatment approaches must be explored to raise treatment outcomes.

Recently, occipital nerve field stimulation (ONS) has been proposed as a potential treatment intervention for fibromyalgia symptoms [8–12]. ONS via a subcutaneous implanted electrode in the area of the greater occipital nerve was initially introduced to treat intractable headache syndromes and can be performed via a minimally invasive procedure [13]. Interestingly, patients with headache disorders comorbid with fibromyalgia showed improvement not only in headaches but also in pain and fibromyalgia-related symptoms [10]. Since then, ONS has been investigated as a potential treatment and seems to offer a safe and effective treatment option for selected medically intractable patients with fibromyalgia.

The occipital nerves interconnect with the trigeminal nerves and form a continuous network affecting the trigeminal nucleus caudalis and the cervical horn at the C1 and C2 levels, which are collectively called as “trigeminocervical complex” [14–16]. Imaging modalities such as functional Magnetic Resonance Imaging (fMRI) and ^18^F-fluorodeoxyglucose Positron Emission Tomography (^18^F-FDG PET) have demonstrated activities in this region of the nervous system during occipital nerve field stimulation [17–20]. An initial fMRI pilot study with a healthy subject undergoing ONS demonstrated that the procedure affects the central nervous system [18]. The predominant areas of activation were in the hypothalamus, thalamus, orbitofrontal cortex, prefrontal cortex, periaqueductal gray area, and cerebellum. A ^18^F-FDG PET study in patients with chronic migraine treated with ONS showed increased activity in the anterior cingulate cortex, pulvinar, and cuneus regions—all of which are involved in the affective dimension of pain [20]. Another ^18^F-FDG PET study on cluster headaches showed that the pregenual anterior cingulate cortex, a major component of the descending pain inhibitory pathway, is involved in the pain-suppressing effects of ONS [17].

The underlying mechanism of fibromyalgia is not known, but there is a possible mechanism contributing to sensitization that relates to increased facilitatory modulation that might go together with dysfunctional inhibitory pathway activity [21]. Two ascending pathways and one descending pathway encode pain from fibromyalgia. The ascending medial pathways encode the motivational/affective components of pain [22, 23], clinically expressed as unpleasantness, while the ascending lateral pathway [23] discriminates the sensory aspects of pain that include localization, intensity, and character of pain. The descending inhibitory pain pathway seems to be involved in decreasing the ongoing pain in a state-dependent manner [24]. Indeed, several neuroimaging studies have shown both structural and functional alterations via connectivity changes that amplify the pain perception in combination with defective inhibition of nociceptive signals [25, 26]. The pregenual anterior cingulate cortex, which plays a critical role in this pain inhibitory pathway, has been found to be altered in fibromyalgia patients [25, 27]. More recent research fine-tunes this concept by considering the underlying pathophysiological mechanism of fibromyalgia as a balance problem between the descending pain inhibitory pregenual anterior cingulate cortex pathway and the pain-detecting dorsal anterior cingulate cortex [19].

The exact mechanism of action that underpins the effect of ONS to treat fibromyalgia-related symptoms is not clear. Therefore, the aim of current study is to investigate how ONS exerts pain inhibition on fibromyalgia patients. Fibromyalgia patients implanted at the greater occipital nerve were scanned using H_2_^15^O – PET to measure regional cerebral blood flow (rCBF). The advantage of using PET instead of fMRI is a reduced risk of electrode migration after implantation along with clearer data that is unaffected by electromagnetic artifacts. In addition, neurophysiological data were also collected. Previous research demonstrated that occipital nerve field tDCS normalizes the imbalance between the pain provoking dorsal anterior cingulate cortex and pain inhibiting pregenual anterior cingulate cortex mainly by modulating the descending pain inhibitory pathway [19]. Based on these previous findings, we expect changes in the descending pain inhibitory pathway during ONS that correlate with a reduction in the pain-related symptoms of fibromyalgia.

## Methods

Patients suffering from fibromyalgia were selected by the Department of Physical Medicine and Rehabilitation at the University Hospital Antwerp, Belgium according to the criteria of the ACR-90 [28]; note that the data were collected in 2010, i.e. before the ACR 2010 guidelines. Patients with pathologies mimicking the symptoms of fibromyalgia as well as patients suffering from severe organic or neuropsychiatric comorbidity (except minor depressive disorder or headache) were excluded from participation. None of the patients were suffering from cervicotrigeminal tract radicular symptoms or types of hemicrania.

All patients enrolled in this study were also part of a large, double blind, placebo-controlled clinical trial that is already published [12]. Seven patients agreed to be part of this sub-study. All patients were female with a mean age of 42.34 years (± 4.53 years). All patients were intractable to tricyclic antidepressants (amitriptyline), pain medication, magnesium supplements, physical therapy, and psychological support. All patients agreed to make no changes to their current medication intake, which primarily included the aforementioned medications. All patients gave written informed consent, and the ethical committee of the University Hospital Antwerp, Belgium approved the study.

### Surgical procedures

The implantation was performed in an operating room under local anesthesia. After removing a small area of the occipital scalp hair, a 2.6-cm vertical incision was made left of the midline just underneath the occipital protuberance. A Tuohy needle was inserted in the subcutaneous plane and tunneled 5.2 cm directed to the contralateral pinna of the ear. Next, a St. Jude Medical Octrode electrode (St Jude Medical, Plano, TX, USA) was inserted through the Tuohy needle, after which the needle was removed. Just underneath the hairline, the lead was tunneled at a sharp angle (315°) to the contralateral side to exit the skin and was then affixed to the skin by a butterfly anchor with a restraining loop. In order to create a similar strain relief loop, the lead was tunneled to a small subcutaneous pocket at the contralateral cervical area in order. In order to connect to an extension lead from the pocket, the lead was tunneled to the ipsilateral intrascapular area (extension 60 cm, St Jude Medical, Plano, TX, USA). The extension lead was tunneled to a subcutaneous pocket in the gluteal area and connected to an internal pulse generator (Eon mini, St. Jude Medical, Plano, TX, USA).

### Stimulation parameters

Patients were stimulated at sub-sensory threshold stimulation for two weeks. This threshold was determined by increasing the amplitude until patients experienced paresthesia and then decreasing the amplitude to 90% of this threshold, with manual pressure overlying the electrode to ascertain no paresthesia would be felt while lying down with pressure on the back of the head.

### Clinical outcomes

The primary outcome parameter for the efficacy of treatment is change in Fibromyalgia Impact Questionnaire scores (FIQ). This questionnaire measures the overall impact of fibromyalgia-related symptoms on a patient’s quality of life. The maximum score is 100, and a higher score indicates a higher disease burden [29]. This questionnaire was assessed at baseline, after 4-weeks, after 12-weeks, after 18-weeks, and after 24-weeks of treatment. The secondary outcomes are the Pain Vigilance and Awareness Questionnaire (PVAQ), Pain Catastrophizing Scale (PCS), and Numeric Rating Scales (NRS) for both pain and quality of life. The PVAQ measures preoccupation with or attention to pain and is associated with pain-related fear and perceived pain severity [30]. The NRS was used to assess quality of life; a higher NRS score indicates a higher quality of life while living with pain caused by a) fibromyalgia, b) bone pain, c) non-specific pain, or d) headache-related pain. It was used to measure symptom relief and treatment satisfaction. This was performed at baseline, after 4-weeks, after 12-weeks, after 18-weeks, and after 24-weeks of treatment.

### Pet

The H_2_^15^O PET scans were acquired with a Siemens Biograph 64 TOF MI PET/computed tomography (Siemens, Knoxville, USA). PET scans were taken during both A) “ON” (active stimulation) and B) “OFF” (stimulation device turned off) conditions. A total of 6 scans (2 conditions × 3 samples) were performed per patient in randomized order. Data acquisition (2 min) started simultaneously with the intravenous bolus injection of 10 mCi H_2_^15^O. There was a 15-min interval between 2 successive injections. The data were reconstructed with the Ordered Subsets Expectation Maximization algorithm followed by a 4-mm Gaussian filter to a 200 × 200 × 74 matrix with zoom set equal to 2 resulting in 2 × 2 × 3 mm voxels.

Image preprocessing was performed using PMOD (version 3.3; PMOD Technologies, Switzerland) and included normalization of the PET images to the SPM water template in MNI space followed by smoothing with a 12-mm FWHM Gaussian Kernel. Voxel-based statistical analysis was carried out using the Statistical Parametric Mapping 8 program (SPM8; Institute of Neurology, University College of London, England, U.K.), implemented in Matlab version 2011a (MathWorks Inc., Natick, MA, USA). The SPM analysis included a flexible factorial design with proportional scaling to account for global changes. Two contrasts were analyzed: (1) ON – OFF (activation) and (2) OFF – ON (deactivation) and the resulting T-map data were interrogated at a peak probability level of 0.05 (uncorrected) and an extent threshold of more than > 250 voxels.

### EEG

Recordings were obtained in a fully lighted room with each participant sitting upright on a small but comfortable chair. The actual recording lasted approximately five minutes. The EEG was sampled using Mitsar-201 amplifiers (NovaTech http://www.novatecheeg.com/) with 19 electrodes placed according to the standard 10–20 International placement (Fp1, Fp2, F7, F3, Fz, F4, F8, T7, C3, Cz, C4, T8, P7, P3, Pz, P4, P8, O1, O2), analogous to what was done in the normative group. Impedances were checked to remain below 5 kΩ. Data were collected eyes-closed (sampling rate = 500 Hz, band passed 0.15–200 Hz). Recordings were done during stimulation (ON) and without stimulation (OFF). We recorded for 2 min ON followed by 2 OFF and continued this pattern until we had 6 min of data for both conditions.

To remove artifacts related to stimulation, we used an ICA method to specifically select the signal related to the stimulation. Neuling and coworkers recently described this method as a reliable way to remove these specific artifacts [31, 32]. In addition, off-line data were resampled to 128 Hz, band-pass filtered in the range 2–44 Hz, subsequently transposed into Eureka! software [33], plotted, and carefully inspected for manual artifact-rejection. All episodic artifacts including eye blinks, eye movements, teeth clenching, body movement, and ECG artifacts were removed from the stream of the EEG. Average Fourier cross-spectral matrices were computed for frequency bands delta (2–3.5 Hz), theta (4–7.5 Hz), alpha1 (8–10 Hz), alpha2 (10–12 Hz), beta1 (13–18 Hz), beta2 (18.5–21 Hz), beta3 (21.5–30 Hz), and gamma (30.5–44 Hz).

Standardized low-resolution brain electromagnetic tomography (sLORETA; Pascual-Marqui, 2002) was used to estimate the intracerebral electrical sources. As a standard procedure, a common average reference transformation [34] was performed before applying the sLORETA algorithm. sLORETA computes electric neuronal activity as current density (A/m^2^) without assuming a predefined number of active sources. The solution space used in this study and associated lead field matrix are those implemented in the LORETA-Key software (freely available at http://www.uzh.ch/keyinst/loreta.htm)*.* This software implements revisited realistic electrode coordinates [35] and the lead field produced by [36] applying the boundary element method on the MNI-152 (Montreal Neurological Institute, Canada). The sLORETA-key anatomical template divides and labels the neocortical (including hippocampus and anterior cingulate cortex) MNI-152 volume into 6239 voxels of dimension 5 mm^3^, based on probabilities returned by the Demon Atlas [37]. The co-registration makes use of the correct translation from the MNI-152 space into the Talairach and Tournoux space.

The methodology used is a non-parametric permutation test. It is based on estimating, via randomization, the empirical probability distribution for the max-statistic under the null hypothesis comparisons [38]. This methodology corrects for multiple testing (i.e. for the collection of tests performed for all voxels and for all frequency bands). Due to the non-parametric nature of this method, its validity does not rely on any assumption of Gaussianity [38]. The significance threshold for all tests was based on a permutation test with 5000 permutations. Comparisons were made between the ON and OFF stimulation conditions. These comparisons were performed on a whole brain by sLORETA statistical contrast maps through multiple voxel-by-voxel comparisons in a logarithm of *t*-ratio.

## Results

### Clinical outcome

Clinical data of patients at baseline, after 4-weeks, after 12-weeks, after 18-weeks, and after 24-weeks of treatment are summarized in Table 1. For the primary outcome measure (FIQ), we observe a decrease of 25.84%. For the secondary outcome measure PVAQ, we found a decrease of 34.51%. For the pain complaints, there was a drop of 30.71% for fibromyalgia pain, 35.75% for bone and joint pain, and 30.52% for non-specified pain. In addition, patients reported a 59.24% improvement in quality of life.

**Table 1:** Primary and secondary outcomes at baseline and 4-weeks, 12-weeks, 18-weeks, and 24-weeks after baseline

	Baseline	4-weeks	12-weeks	18-weeks	24-weeks	*p* value
*Primary Outcome Measure*						
FIQ	59.26^a^	37.16^b^	40.36^b^	42.04^b^	43.95^b^	0.006
*Secondary Outcome Measures*						
PVAQ	40.57^a^	32.71^b^	26.71^b^	26.71^b^	26.57^b^	0.003
PCS	20.84^a^	10.43^b^	8.86^b^	8.29^b^	10..28^b^	0.006
NRS						
Overall Quality of Life	3.14^a^	6.00^b^	6.14^b^	6.00^b^	5.00^b^	0.01
Overall Fibromyalgia Pain	7.00^a^	4.00^b^	4.29^b^	4.42^b^	4.85^b^	0.001
Overall Bone and Joint Pain	8.00^a^	4.14^b^	5.14^b^	4.86^b^	5.14^b^	0.004
Overall Non-Specified Pain	5.57^a^	4.00^a,b^	3.28^b^	3.42^b^	3.87^b^	0.033

^a, b^ indicate that they are significantly different

### PET results

**Fig. 1:**
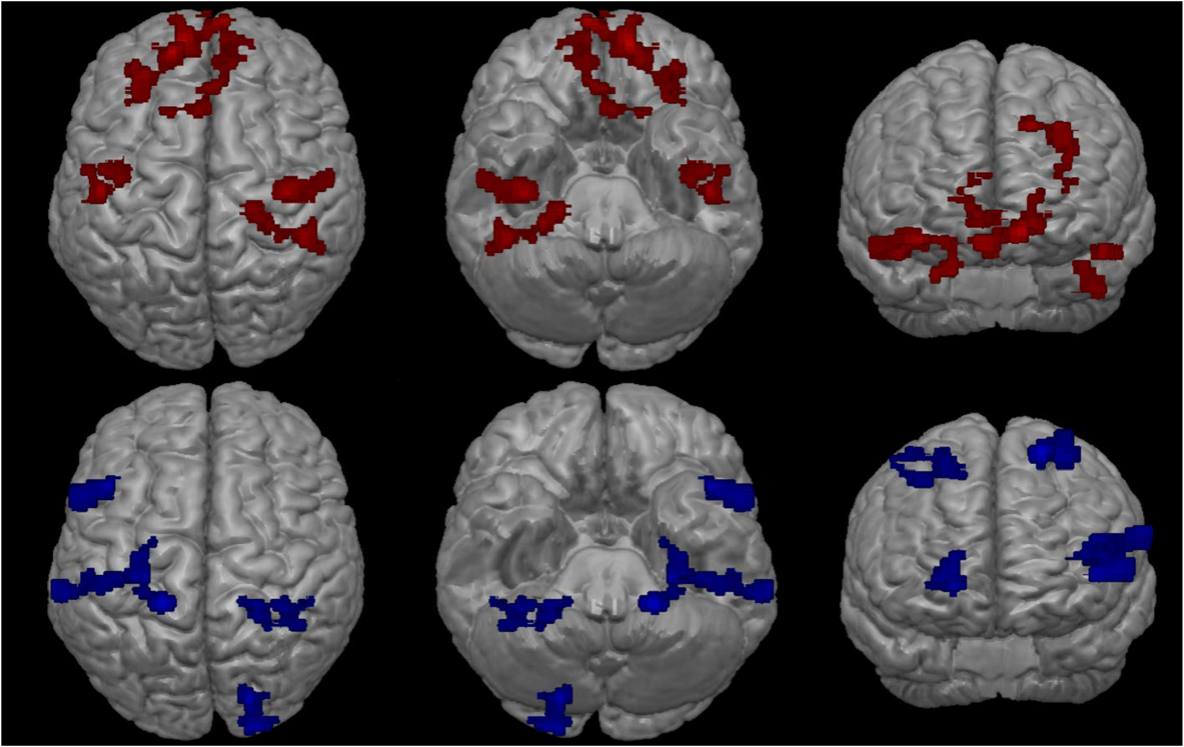
Pet scan data regions of activation (on - off; red) and regions of deactivation (off - on; blue)

We identified activation (ON – OFF contrast) in the left ventral medial prefrontal cortex, the dorsal lateral prefrontal cortex, the left superior frontal gyrus, the right parahippocampal gyrus, the left inferior temporal gyrus extending into the right fusiform gyrus, and the bilateral anterior cingulate cortex. In addition, deactivation (OFF – ON contrast) was demonstrated in the left somatosensory association cortex, the right lingual gyrus extending into the cuneus, the left precentral gyrus, the left supramarginal gyrus, and the right precuneus (See Table 2 and Fig. 1).

**Table 2:** Statistical Parametric Mapping PET Analysis: regions of activation (ON – OFF) and regions of deactivation (OFF – ON) with *p*_uncorrected_ < 0.05 and k > 250 voxels

Region	Talairach coordinates (x, y, z)			Side	Area	*t*-value
Activation	−11.80	49.47	0.25	Left	Ventral medial prefrontal cortex	5.27
	−16.18	41.41	43.96	Left	Dorsolateral prefrontal cortex	4.41
	34.29	−17.32	−11.20	Right	Parahippocampus	4.06
	−45.22	−16.39	−17.86	Left	Left inferior temporal gyrus	3.51
	39.86	−39.19	−18.58	Right	Fusiform gyrus	3.51
	0.93	28.21	5.16	Interhemispheric	Pregenual anterior cingulate cortex	3.21
Deactivation	−20.35	−42.47	55.76	Left	Somatosensory cortex	4.60
	17.30	−81.81	−4.98	Right	Visual cortex	3.89
	16.90	6.97	3.88	Left	Ventral lateral prefrontal cortex	3.88
	−62.46	−35.21	21.50	Left	Auditory cortex	3.01
	18.56	−44.19	52.65	Right	Precuneus	2.99

### EEG results

**Fig. 2:**
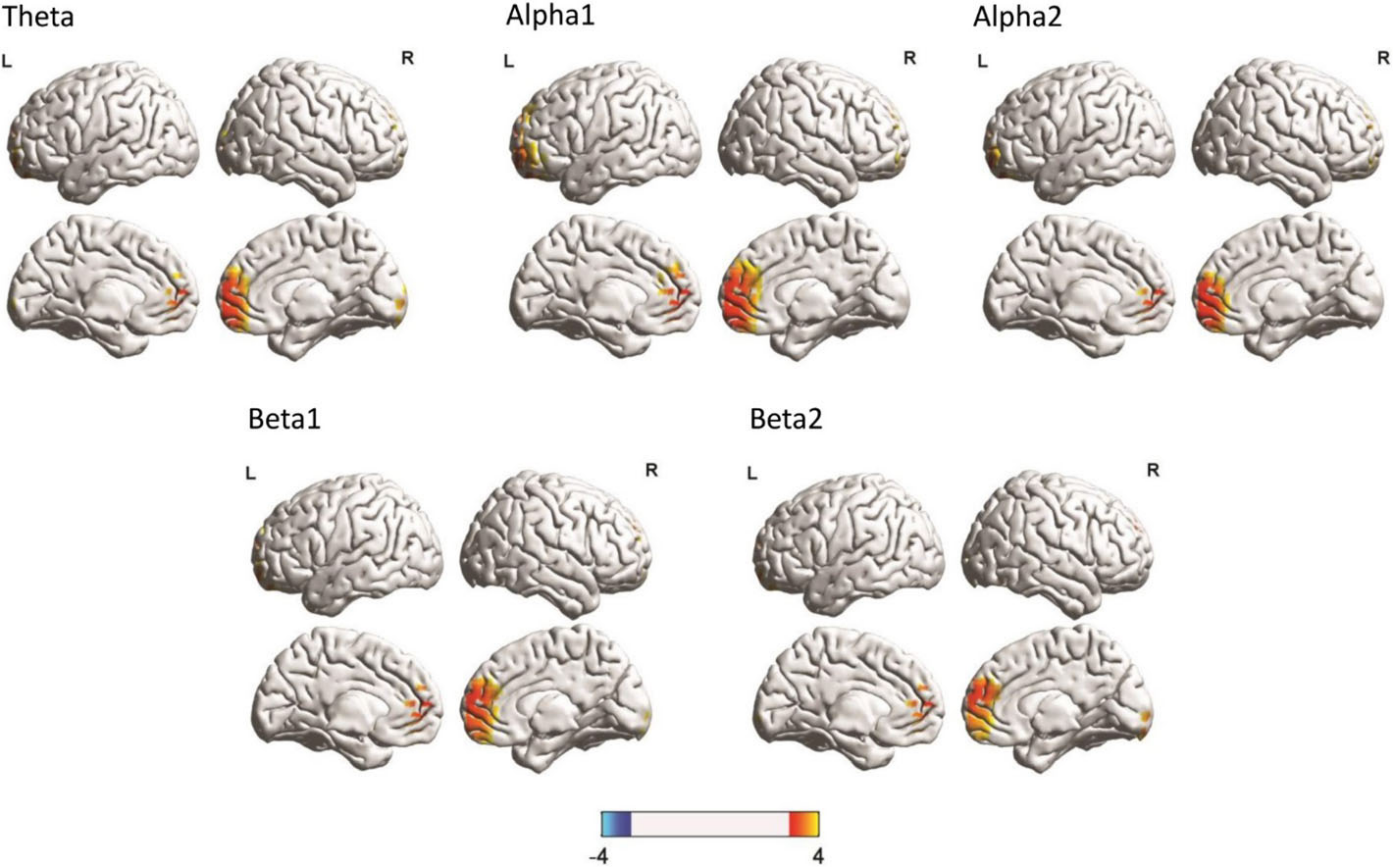
A comparison between the on and off stimulation conditions show a significant increase in activity at the pregenual anterior cingulate cortex extending into the ventral medial prefrontal cortex for the theta, alpha1, alpha2, beta1, and beta2 frequency bands during stimulation

A comparison between the ON and OFF stimulation conditions shows a significant increase (*t* = 3.65, *p* <. 05) in activity at the pregenual anterior cingulate cortex extending into the ventral medial prefrontal cortex for the theta, alpha1, alpha2, beta1, and beta2 frequency bands during stimulation (see Fig. 2). No effects were obtained for the delta, beta3, or gamma frequency bands.

## Discussion

This study aims to better understand the underlying neural effects of occipital nerve field stimulation (ONS) for the treatment of fibromyalgia using H_2_^15^O PET. The therapeutic outcome after 24 weeks of treatment was similar to the outcome in a larger population showing significant decreases in both the perceptual and affective components of pain as well as an improvement in quality of life [12]. Our research demonstrated increased rCBF changes (activation) in the ventral medial prefrontal cortex, dorsal lateral prefrontal cortex, pregenual anterior cingulate cortex, and parahippocampus. In addition, we also observed decreased rCBF (deactivation) in the somatosensory cortex, the ventral lateral prefrontal cortex, and the precuneus during subthreshold stimulation in comparison to no subthreshold stimulation. We also found increased activity in the pregenual anterior cingulate cortex extending into the ventral medial prefrontal cortex for the theta, alpha1, alpha 2, beta1, and beta2 frequency bands during subthreshold stimulation in comparison to no subthreshold stimulation. In the discussion, we will only focus on the PET findings.

Our results showed increased activity for both H_2_^15^O PET and EEG in the pregenual anterior cingulate cortex extending into the ventral medial prefrontal cortex during ONS in comparison to no stimulation. The pregenual anterior cingulate cortex extending into the ventral medial prefrontal cortex, as well as the periaqueductal gray, parahippocampus, anterior insula, hypothalamus, and rostral ventromedial brainstem are all part of the descending pain inhibitory or antinociceptive pathway [24, 39]. Previous research has shown that this descending pathway is involved in stress-mediated pain inhibition [40] and placebo analgesia [41] and is deficient in pain syndromes such as fibromyalgia [42]. This deficiency in the descending pain pathway could explain why fibromyalgia patients have spontaneous widespread pain all over their body, i.e. the pain results from insufficient spontaneous pain suppression, and is non-topographic. Mechanistically, the rostral- to pregenual anterior cingulate cortex is functionally connected to the periaqueductal gray [43], and this resting state functional connectivity between the anterior cingulate cortex and the periaqueductal gray is abnormal in fibromyalgia patients [44]. Furthermore, a direct link between the pregenual anterior cingulate cortex and periaqueductal gray (i.e. main areas of the descending pain pathways) and the C2 area has been shown [45]. This fits with our findings that stimulating the greater occipital nerve modulates the pregenual anterior cingulate cortex extending into the ventral medial prefrontal cortex. The changes seen in the pregenual anterior cingulate cortex activity both on PET and EEG functional imaging during ONS are associated with a decrease in pain complaints. It is therefore very likely that ONS reverses the dysfunction of the pain inhibition pathway in fibromyalgia, resulting in the pregenual anterior cingulate cortex extending into the ventral medial prefrontal cortex regaining its ability to suppress pain. A recent PET study in patients with cluster headache showed that changes in the pregenual anterior cingulate cortex are involved in the pain-suppressing effect of ONS [46]. This suggests that the pregenual anterior cingulate cortex is involved in pain suppression in a non-specific way, as a similar mechanism is involved in migraine and fibromyalgia related pain.

Increased activity was also observed for the left dorsal lateral prefrontal cortex during ONS in comparison to no stimulation using H_2_^15^O PET. Previous research already demonstrated the involvement of the dorsal lateral prefrontal cortex in cognitive processes [47] such as attention [48, 49], value encoding [50–52], and emotional regulation [53]. Important to the concept of pain, the left dorsal lateral prefrontal cortex has also been associated with regulation of top-down modulation and driving appropriate behavioral responses [54, 55]. Indeed, a recent PET study reported that the dorsal lateral prefrontal cortex plays a role in inhibiting pain [56]. Furthermore, it has been reported that stimulating the left dorsal lateral prefrontal cortex using non-invasive brain stimulation improves attention in patients with cognitive dysfunction. Taken together, these findings lead to the hypothesis that left dorsal lateral prefrontal cortex activation during stimulation inhibits the affective/emotional pain pathway via a top-down mechanism. Other supporting evidence for this effect comes from spinal cord stimulation (SCS) for pain. It has been shown that burst SCS, in contrast to tonic SCS, modulates the affective/motivational/attentional component of pain [57–60], and this is associated with changes in the dorsal anterior cingulate cortex and dorsal lateral prefrontal cortex [58].

Our results also demonstrate increased activation of the parahippocampus during ONS. The parahippocampus is associated with contextual processing and is important in pain processing [61–65]. It has been shown that fibromyalgia is associated with metabolite abnormalities within the right (para)hippocampus that correlate with patient symptoms due to chronic stress [66, 67]. This is consistent with the notion of a generalized aversion/distress network consisting of the parahippocampus, cerebellum, hypothalamus, and subgenual anterior cingulate cortex [68]. Parahippocampal involvement in pain might be due to contextual memory, which can modulate pain via its influence on the descending pain pathway, thereby encoding aversive pain memory [39, 69]. In fibromyalgia, it is known that this contextual pain suppression mechanism is dysfunctional, leading to a subsequent dysregulation of emotional contextual pain suppression [42, 70]. Based on these findings it is hypothesized that the parahippocampal area is a control switch for the involvement of the ascending medial pathway in the affective component of pain and the descending pain inhibitory pathway [71]. Conceptually, stimulation of the greater occipital nerves activates the parahippocampus, which subsequently controls the mobilization of the medial pain and descending inhibitory pathway [72].

Although several papers have reported activity changes in the inferior temporal and fusiform gyri in the pain literature, the exact functional significance of these changes is unknown. It has been hypothesized that these areas are involved in cognitive pain-processing related to greater vigilance and attention to pain. This is based on recollection of prior pain experience, expectations for future pain, and negative pain appraisal [73]. Interestingly, both anxiety and mental fatigue have been negatively associated with activation of the inferior temporal gyrus/fusiform gyrus [74]. Our data show increased activation during ONS in the inferior temporal and fusiform gyri might be due to reduced anxiety and/or mental fatigue; however, further research is needed to verify this speculation.

We also observed decreased rCBF in the somatosensory, auditory, and visual cortices during ONS in comparison to no stimulation. Previous literature has already suggested an indirect connection between the C2-C3 nerve and these sensory areas [75, 76], and occipital nerve stimulation has been shown to help in the suppression of auditory phantom percepts such as tinnitus [77–79]. The role of the somatosensory cortex as part of the lateral pain pathway responsible for encoding discriminatory/sensory pain information is well known in the pain literature generally [80], but even more specifically in fibromyalgia [81–84]. Our findings suggest that ONS can also modulate the lateral pain pathway, thereby changing the balance between pain input and pain suppression [85], leading to probable restoration of defective intracortical inhibition as we hypothesized [86]. This also confirms a recent study using functional magnetic resonance imaging during ONS that shows alterations in the somatosensory cortex during stimulation in healthy subjects [18].

Another interesting finding is reduced rCBF in the precuneus when the stimulation is turned on. The precuneus extending into posterior cingulate cortex is thought to comprise the functional core of the default mode network, which also includes the bilateral inferior parietal cortices and medial prefrontal cortex. It is known that the default mode network undergoes reorganizational changes and functional connectivity changes during chronic pain in fibromyalgia patients [87]. The default mode network is activated when attention is engaged with thoughts unrelated to pain or mind wandering and deactivated when the attention is focused on pain [88]. Furthermore, it is known that the precuneus extending into the posterior cingulate cortex is activated during pain processing and inactive during (placebo) analgesia [89, 90]. Similarly, ONS reduces rCBF in the precuneus extending into the posterior cingulate cortex in fibromyalgia, leading to pain suppression. Alternatively, it also possible that ONS modifies the pain percept and indirectly modulates the internal model (self-reference) of pain that is associated with fibromyalgia patients. The main hub of the self-referential default mode network is the posterior cingulate cortex [91, 92] that allows adaptation to changes in the environment [93]. Adapting to these changes requires that internal and external stimuli are predicted and compared to the current state of the self. This likely occurs at the posterior cingulate cortex [94–96]. ONS consequently could induce reference resetting due to changes in the precuneus extending into the posterior cingulate—areas of the brain that could obtain an internal reference without pain, analogous to what has been proposed in obesity. Indeed, in food addiction, it has been proposed that the self-referential set point of how much energy was required to maintain a stable energetic milieu interieur critically depends on the posterior cingulate/precuneus, and that the precuneus resets the balance between food input (i.e. dorsal anterior cingulate cortex) and food input suppression (pregenual anterior cingulate cortex) [97]. Even though this is speculation, it is possible that the posterior cingulate cortex’s function, as a regulator of the body’s adaptation to the external and internal environment, might indeed be analogous for different stimuli, i.e. might be non-specific, similar to the dorsal anterior cingulate cortex, whether for pain or for food.

## Conclusion

In this study, we demonstrated the effect of ONS in fibromyalgia patients and specifically its effect on different brain structures. ONS seems to exert an inhibitory effect on structures in the ascending lateral pathways of multiple sensory inputs (such as the somatosensory, visual, and auditory cortices) and medial pain pathways (such as the dorsal lateral prefrontal cortex) that mediate the affective component of pain. ONS also modulates the descending pain pathway by activating the pain-suppressing pregenual anterior cingulate cortex and parahippocampal area. Further functional imaging studies should be performed to evaluate these findings on a larger sample size and with other neuromodulation techniques.

## Abbreviations

^18^F-FDG PET: ^18^F-fluorodeoxyglucose positron emission tomograph
EEG: Electroencephalogram
FIQ: Fibromyalgia impact questionnaire scores
fMRI: Functional magnetic resonance imaging
NRS: Numeric rating scales
ONS: occipital nerve field stimulation
PCS: Pain catastrophizing scale
PVAQ: Pain vigilance and awareness questionnaire
rCBF: Regional cerebral blood flow
SCS: Spinal cord stimulation
